# Author Correction: Hydrophobic pore gates regulate ion permeation in polycystic kidney disease 2 and 2L1 channels

**DOI:** 10.1038/s41467-019-09422-4

**Published:** 2019-03-26

**Authors:** Wang Zheng, Xiaoyong Yang, Ruikun Hu, Ruiqi Cai, Laura Hofmann, Zhifei Wang, Qiaolin Hu, Xiong Liu, David Bulkley, Yong Yu, Jingfeng Tang, Veit Flockerzi, Ying Cao, Erhu Cao, Xing-Zhen Chen

**Affiliations:** 1 National “111” Center for Cellular Regulation and Molecular Pharmaceutics, Hubei University of Technology, Wuhan, Hubei 430068 China; 2 Department of Physiology, Membrane Protein Disease Research Group, Faculty of Medicine and Dentistry, University of Alberta, Edmonton, AB T6G 2H7 Canada; 3 Department of Biochemistry, University of Utah School of Medicine, Salt Lake City, UT 84112 USA; 4 School of Life Sciences and Technology, Tongji University, Shanghai, 200092 China; 5 Experimentelle und Klinische Pharmakologie und Toxikologie, Universität des Saarlandes, Homburg, 66421 Germany; 6 Department of Biological Sciences, St. John’s University, Queens, NY 11439 USA; 7 Keck Advanced Microscopy Laboratory and Department of Biochemistry and Biophysics, University of California, San Francisco, San Francisco, CA 94143 USA

Correction to: *Nature Communications* 10.1038/s41467-018-04586-x, published online 13 June 2018

The original version of this Article contained an error in the spelling of the author David Bulkley, which was incorrectly given as David Bulkey. This has now been corrected in both the PDF and HTML versions of the Article.

