# Supplementary material for: Hydrophobic pore gates regulate ion permeation in polycystic kidney disease 2 and 2L1 channels
Source: Nat Commun. 2018 Jun 13;9:2302. doi: 10.1038/s41467-018-04586-x (PMC5998024; doi:10.1038/s41467-018-04586-x)
Supplement: Supplementary file 1 — Supplementary Information [file 41467_2018_4586_MOESM1_ESM.pdf]

Supplementary Information

Hydrophobic pore gates regulate ion permeation in polycystic kidney disease 2 and 2L1 channels

Zheng et al.

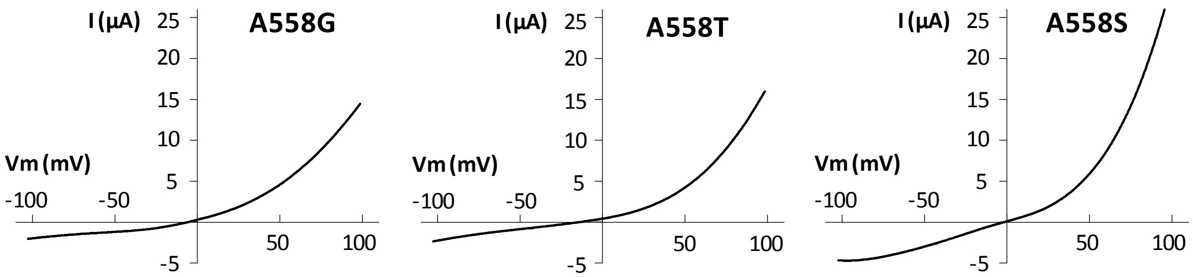

Supplementary Figure 1. Representative I-V curves for PKD2L1 A558G, A558T and A558S mutants. Currents were recorded in expressing oocytes in the presence of the Mg^{2+}-free Na-containing solution (in mM): 100 NaCl, 2 KCl, 10 HEPES at pH 7.5.

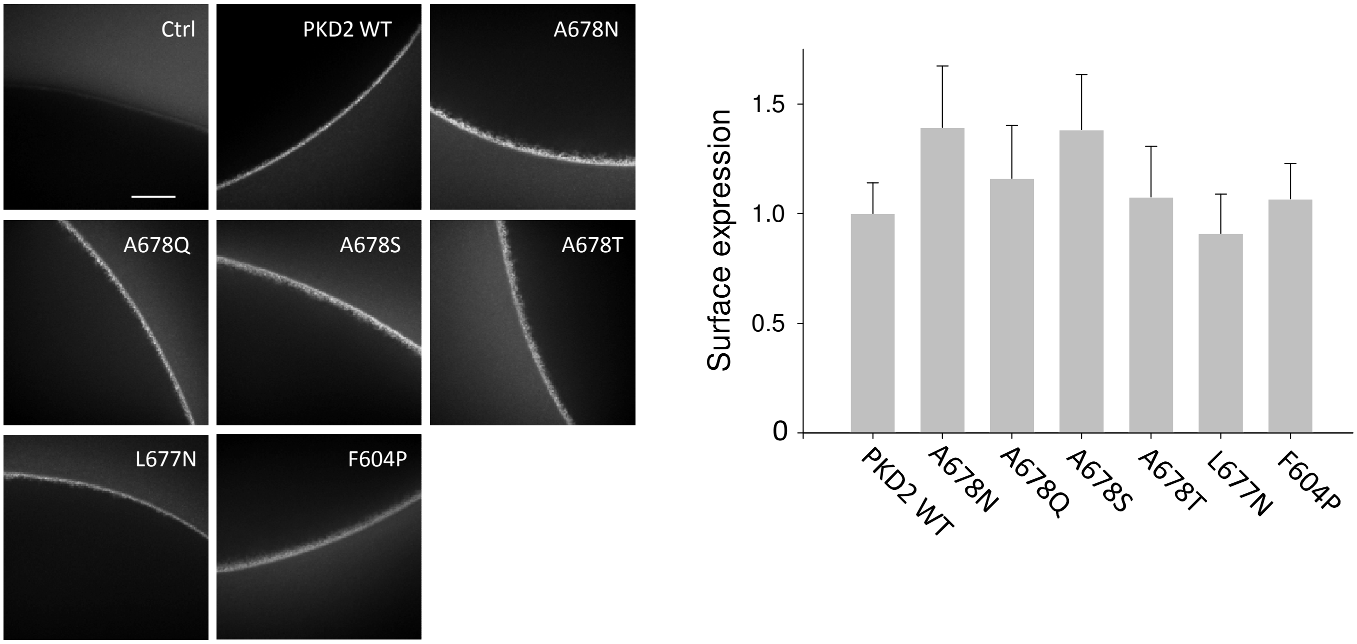

Supplementary Figure 2. Surface expression of WT or mutant PKD2 in oocytes. Left panel, whole-mount immunofluorescence using the antibody for the HA-tag showing the oocyte surface expression of the PKD2 WT or indicated mutants; Ctrl, water injected oocytes. Scale bar, 50 µm. Right panel, averaged surface membrane expression of the PKD2 mutants normalized to that of PKD2 WT (n = 4 - 7). Data are presented as mean ± SEM.

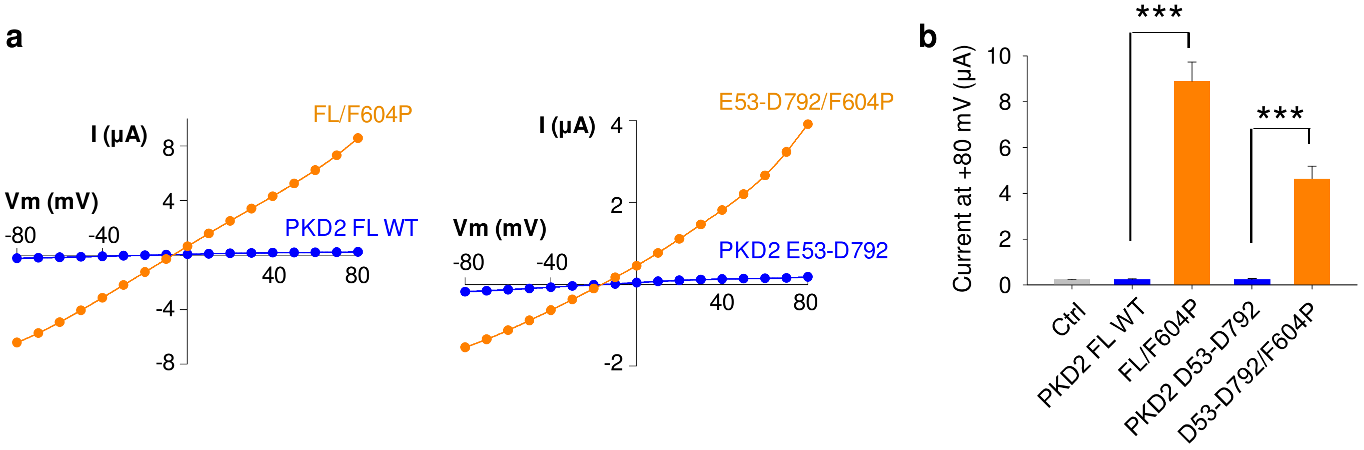

Supplementary Figure 3. Characterization of human PKD2 truncated mutant E53-D792. a Representative I-V curves of full-length (FL) wild-type (WT) PKD2 and truncated mutant E53-D792 without or with F604P point mutation obtained under the same experimental condition as in Fig. 3a. **b** Averaged currents at +80 mV in expressing or water-injected (Ctrl) oocytes, as indicated. Data are presented as mean ± SEM. ***$P < 0.001$ by Student's t-test.

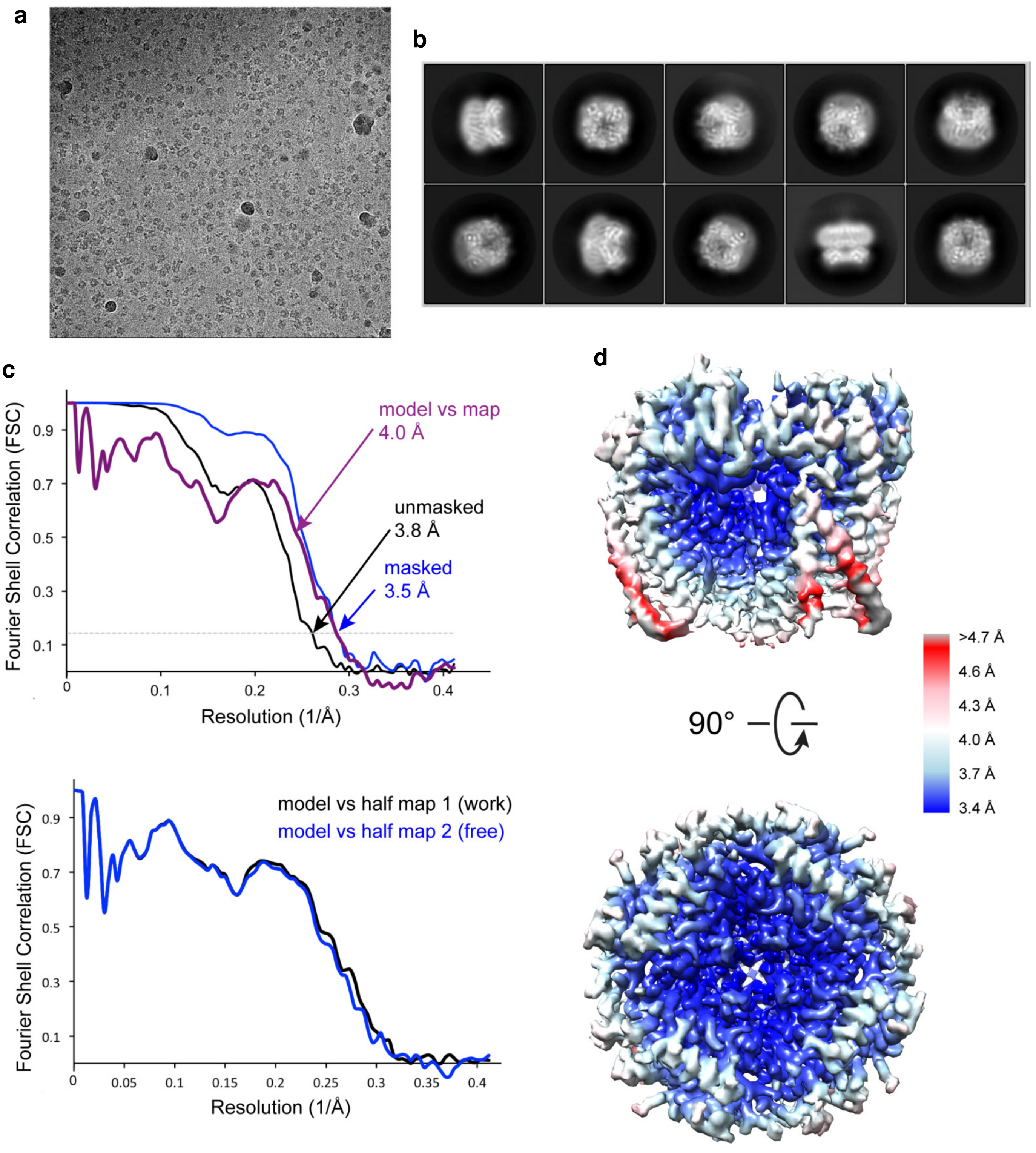

Supplementary Figure 4. Structure determination of the PKD2 F604P channel. a A representative micrograph of PKD2 F604P particles recorded on a TF30 Polara microscope. **b** 2D classes of PKD2 F604P calculated with RELION. **c** FSC curves for resolution estimation and model validation. (top) FSC curves reported by RELION before (unmasked, black) and after (masked, blue) postprocessing, where an automask was generated by RELION to flattening regions outside protein density prior to FSC calculation. FSC curves (model vs summed half map, purple) show overall agreement between model and map. (bottom) FSC curves are calculated for model vs half map 1 (work, black) and model versus half map 2 (free, blue). Overall agreement between these curves indicates little over-fitting of the model. **d** Local resolution determined by RELION.

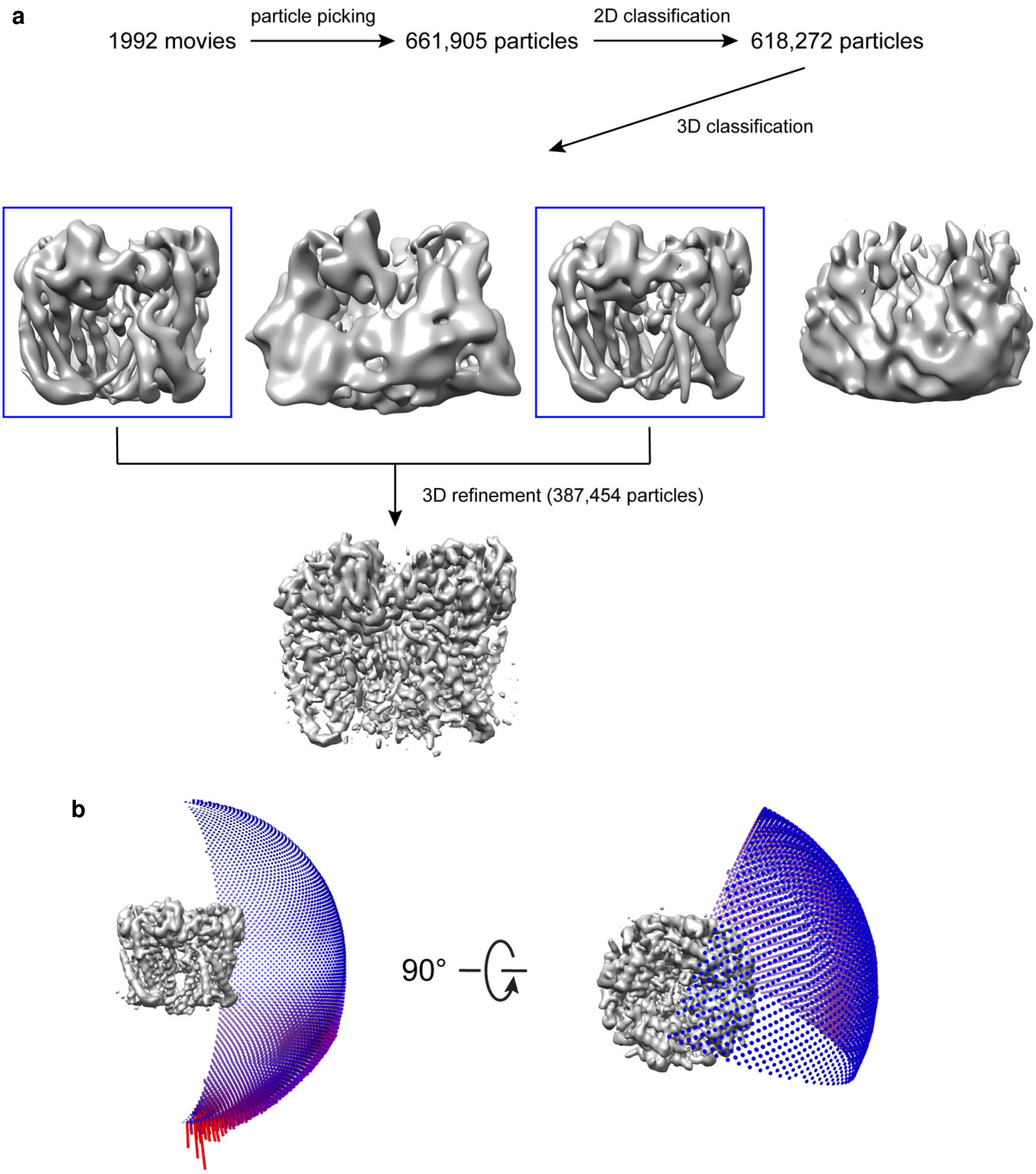

Supplementary Figure 5. Reconstruction of the PKD2 F604P channel with C4 symmetry. **a** Flow chart of image processing for the PKD2 F604P channel with C4 symmetry imposed. **b** Angular distribution plot of all particles projections. Cylinder heights are proportional to number of particles assigned to each set of Euler angles.

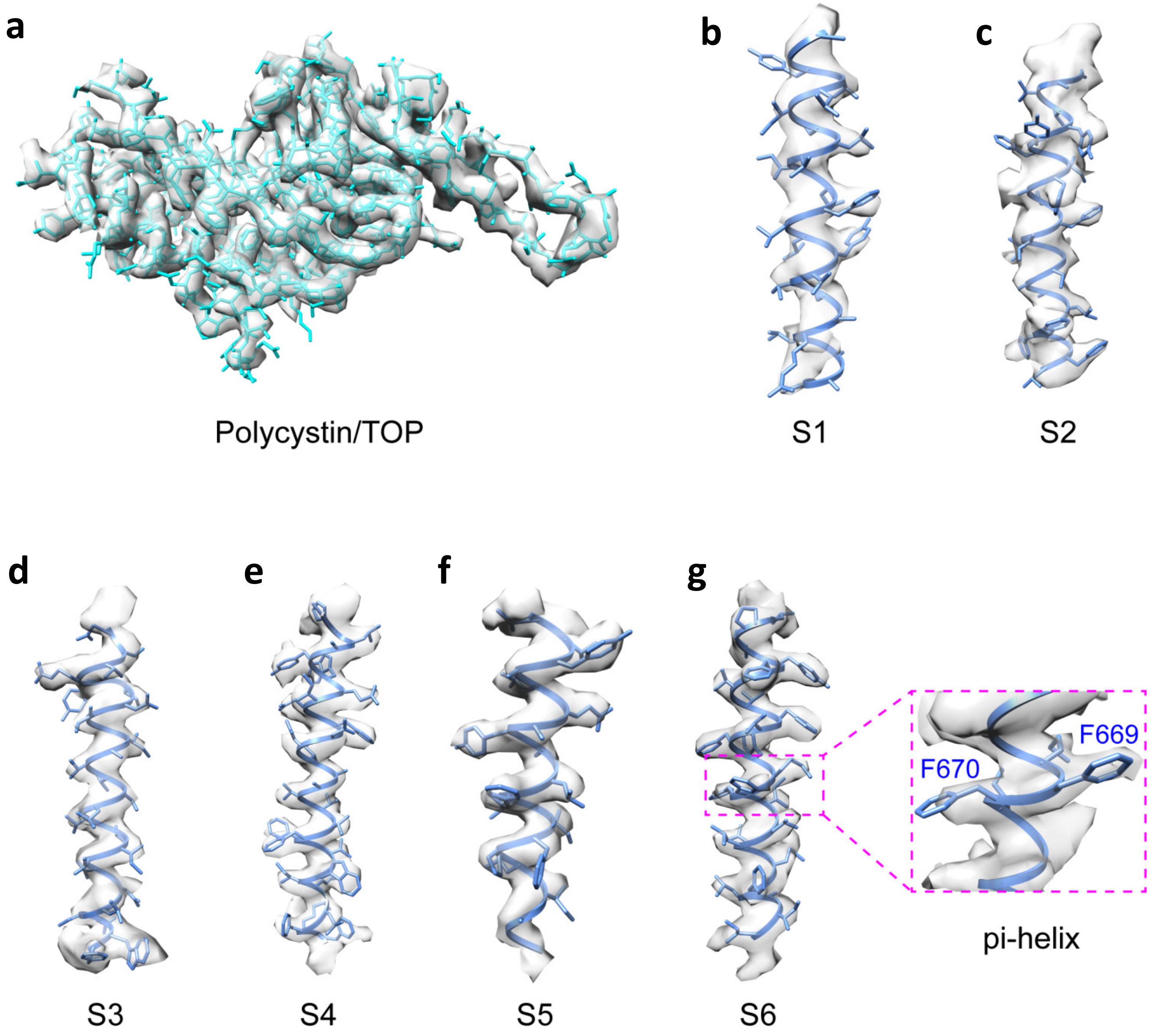

Supplementary Figure 6. EM density maps of various regions of the PKD2 F604P channel. The maps are sharpened with a b factor of -150 Å^2. In panel G, the pi-helix is highlighted.

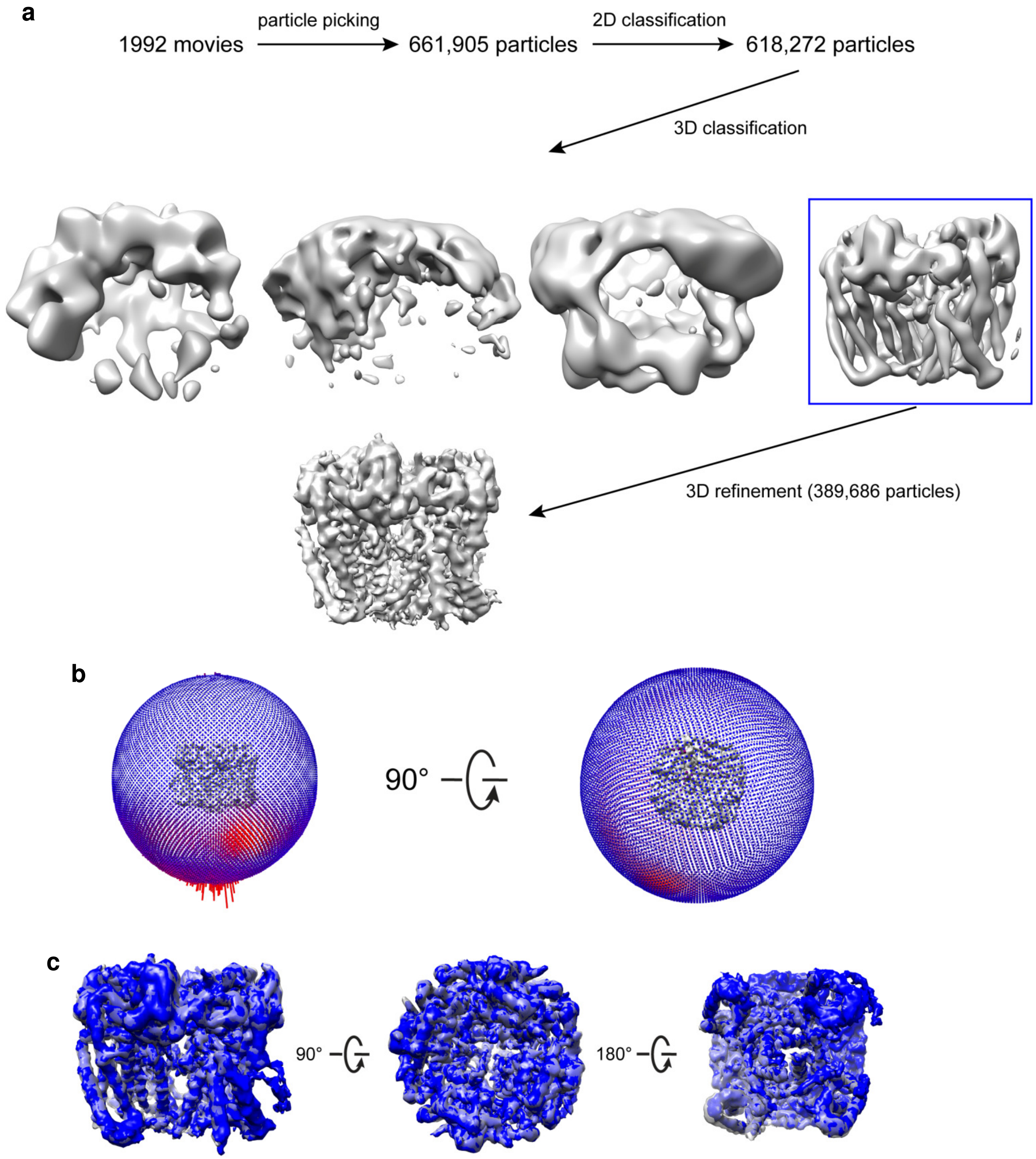

Supplementary Figure 7. Reconstruction of the PKD2 F604P channel without C4 symmetry. a Flow chart of image processing for the PKD2 F604P channel without C4 symmetry imposed. **b** Angular distribution plot of all particles projections. Cylinder heights are proportional to number of particles assigned to each set of Euler angles. **c** Comparison of the maps reconstructed with (gray) or without (blue) C4 symmetry, showing their overall agreement. Only subtle differences within the peripheral S1 helix, flexible loops, and amphipols are observed. Overall, map determined without C4 symmetry is of lower resolution as compared with the map determined with C4 symmetry. In particular, the S4-S5 linker is not welled resolved in the map without imposing C4 symmetry.

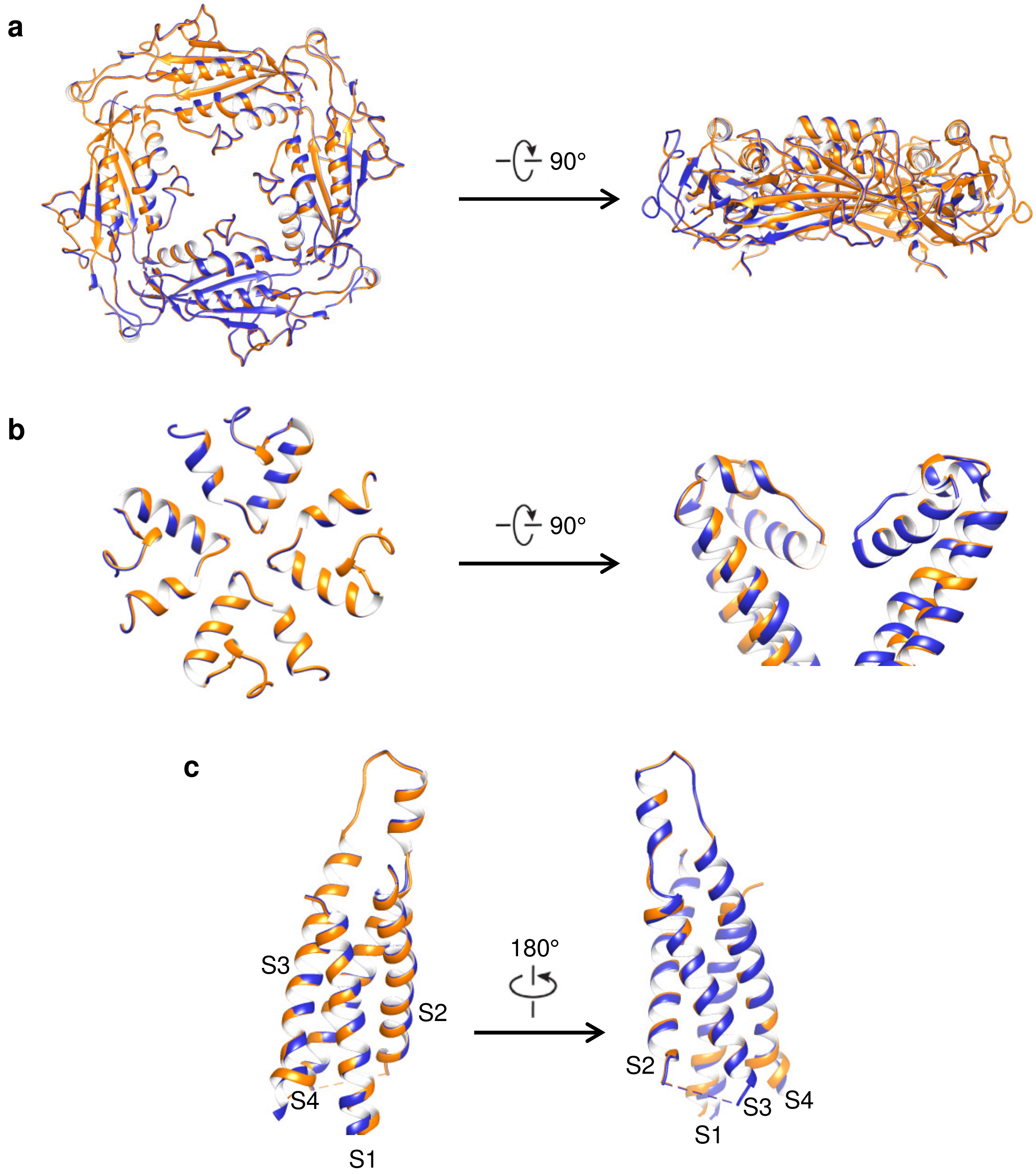

Supplementary Figure 8. Superposition of PKD2 WT and F604P mutant structures.
Superposition of TOP domains (**a**), selectivity filters (**b**) and S1-S4 helices (**c**). PKD2 WT, blue; F604P, orange.

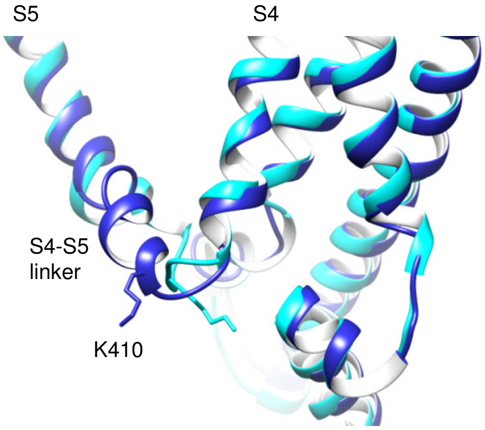

Supplementary Figure 9. Comparison between the TRPML1 S4-S5 linker in the closed (blue, PDB: 5WPQ) and proposed pre-open (cyan, PDB: 5WPT) states. The K410 in the linker's N-terminus, corresponding to the R581 in PKD2, is indicated.

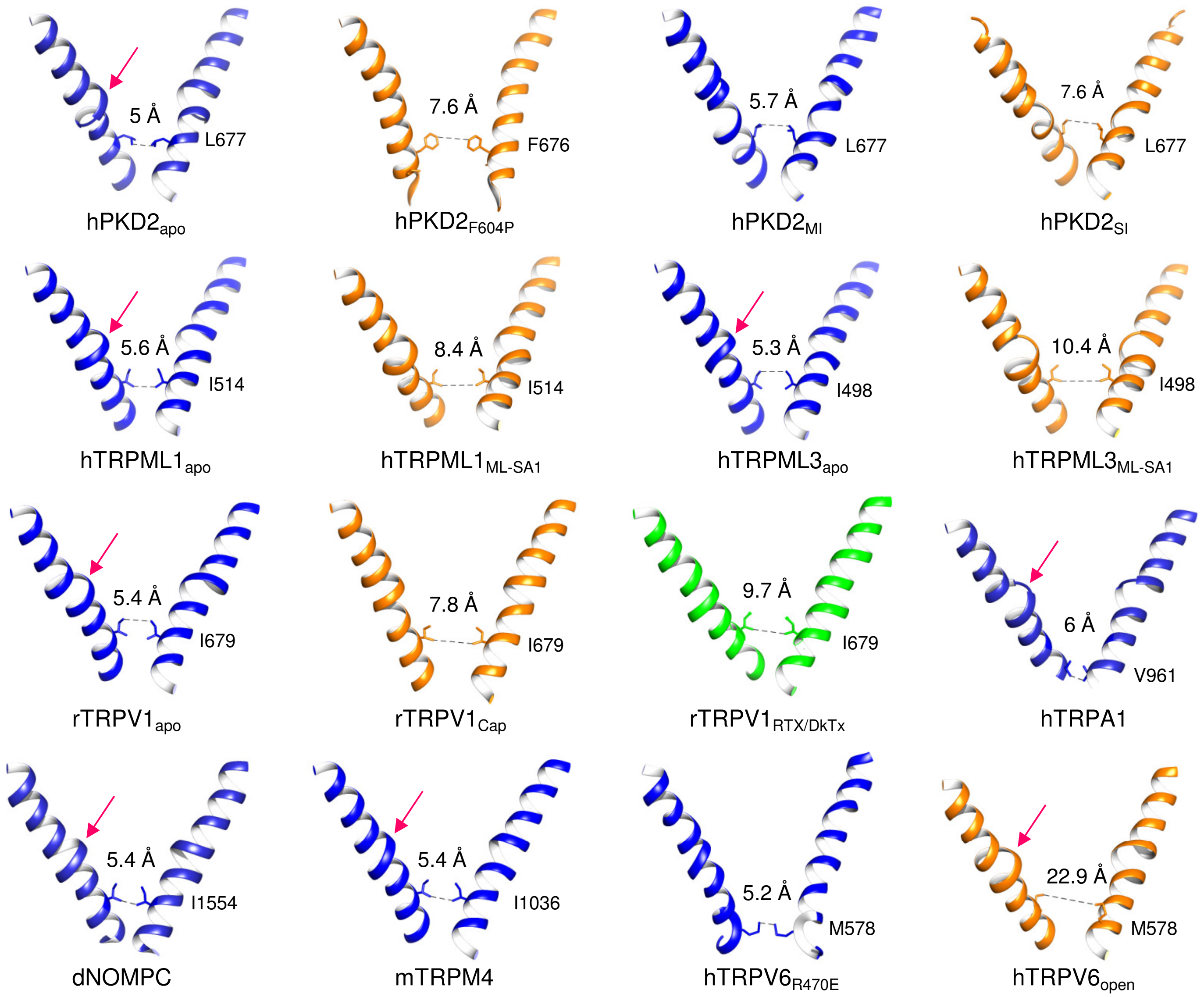

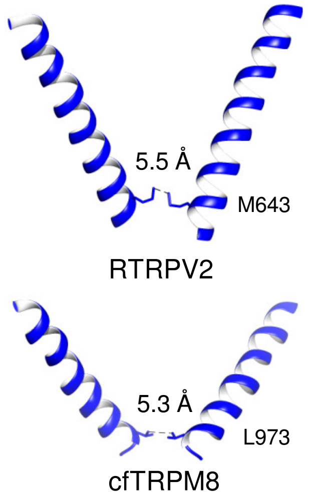

	S6	
hPKD2	GPIYFTTFVFF**MFFIL**LNMFLAIIND	(682)
hTRPML1	SQLYLYSFISL**FIYMV**LSLFIALITG	(519)
hTRPML3	SRIYLYSFISL**FIYMI**LSLFIALITD	(503)
rTRPV1	FIILLLAYVIL**TYILL**LNMLIALMGE	(684)
hTRPA1	SFAQLVSFTIF**VPIVL**MNLLIGLAVG	(962)
dNOMPC	FKIVFGIYMLV**SVVVL**INLLIAMMSD	(1559)
mTRPM4	VVLLLIVFLLV**ANILL**LNLLIAMFSY	(1041)
hTRPV6	YSITYAAFAII**ATLLM**LNLLIAMMGD	(580)
RTRPV2	VLLLLLAYVLLTYVLLLNMLIALMSE	(645)
cfTRPM8	TIPLVCIYMLSTNILLVNLLVAMFGY	(980)

Supplementary Figure 10. π-helix and α-helix in S6 of TRP channels. Upper and lower left panel, pore-lining S6 helices in $hPKD2_{apo}$ (apo state, PDB: 5T4D), $hPKD2_{MI}$ (bound with multiple Ca^{2+} ions, PDB: 5MKF), $hPKD2_{SI}$ (bound with single Ca^{2+} ion, PDB: 5MKE), $hTRPML1_{apo}$ (PDB: 5WJ5), $hTRPML1_{ML\text{-}SA1}$ (bound with agonist ML-SA1, PDB: 5WJ9), $hTRPML3_{apo}$ (PDB: 6AYE), $hTRPML3_{ML\text{-}SA1}$ (bound with ML-SA1, PDB: 6AYF), $rTRPV1_{apo}$ (PDB: 3J5P), $rTRPV1_{Cap}$ (bound with capsaicin, PDB: 3J5R), $rTRPV1_{RTX/DkTx}$ (bound with RTX/DkTx, PDB: 3J5Q), hTRPA1 (PDB: 3J9P), dNOMPC (PDB: 5VKQ), mTRPM4 (PDB: 6BCJ), $hTRPV6_{R470E}$ (full-length human TRPV6 with the R470E mutation, PDB: 6BOA), $hTRPV6_{open}$ (full-length human TRPV6, PDB: 6BO9), RTRPV2 (PDB: 5AN8), cfTRPM8 (PDB: 6BPQ). The gate residues revealed by the structures are shown and the pore diameters determined by the gate residues indicated. The π-helix in the middle of S6 is indicated by a red arrow. h, human; r, rat; d, *drosophila* (fruit fly); m, mouse; R, rabbit; cf, collared flycatcher. Lower right panel, amino acid sequence alignment of S6 helices from hPKD2 (Universal Protein Resource accession #: Q13563), hTRPML1 (Q9GZU1), hTRPML3 (Q8TDD5), rTRPV1 (O35433), hTRPA1 (O75762), dNOMPC (Q7KIQ2), mTRPM4 (Q7TN37), hTRPV6 (Q9H1D0), RTRPV2 (G1SNM3), cfTRPM8 (U3JD03). The last amino acid numbers are indicated and the π-helix sequences are highlighted bold and magenta.

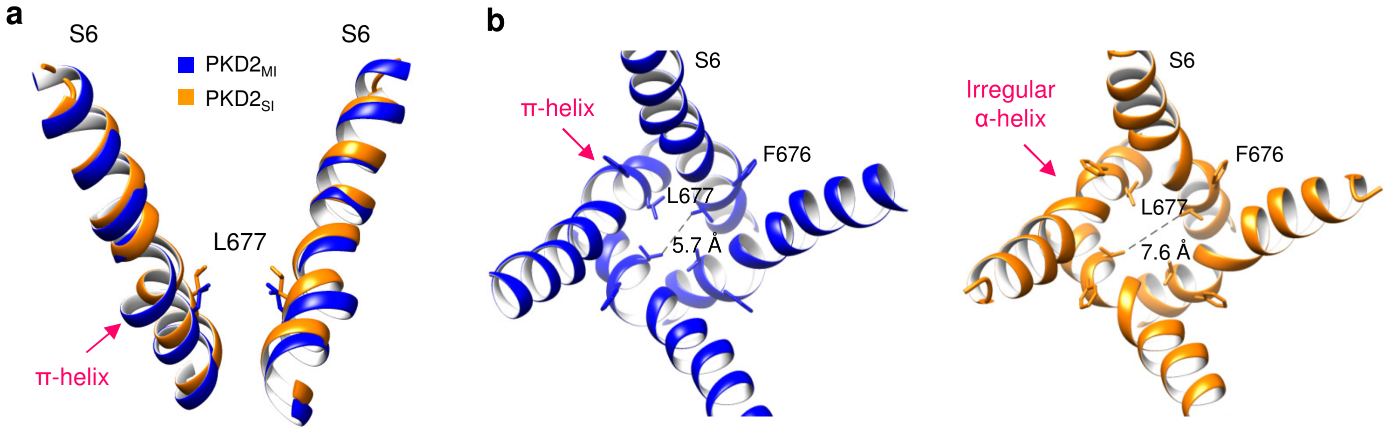

Supplementary Figure 11. Conformational changes in pore-lining S6 helix of PKD2 induced by Ca^{2+}. **a** Side view of superposition of S6 in the $PKD2_{MI}$ (bound with multiple Ca^{2+} ions, PDB: 5MKF, blue) and $PKD2_{SI}$ (bound with single Ca^{2+} ion, PDB: 5MKE, orange). The π-helix in the $PKD2_{MI}$ structure is indicated by an arrow. The gate residue L677 is shown. **b** Top view of the pore lined by S6 in the $PKD2_{MI}$ and $PKD2_{SI}$ structures showing local conformational changes in the middle of S6.

Figure 1e

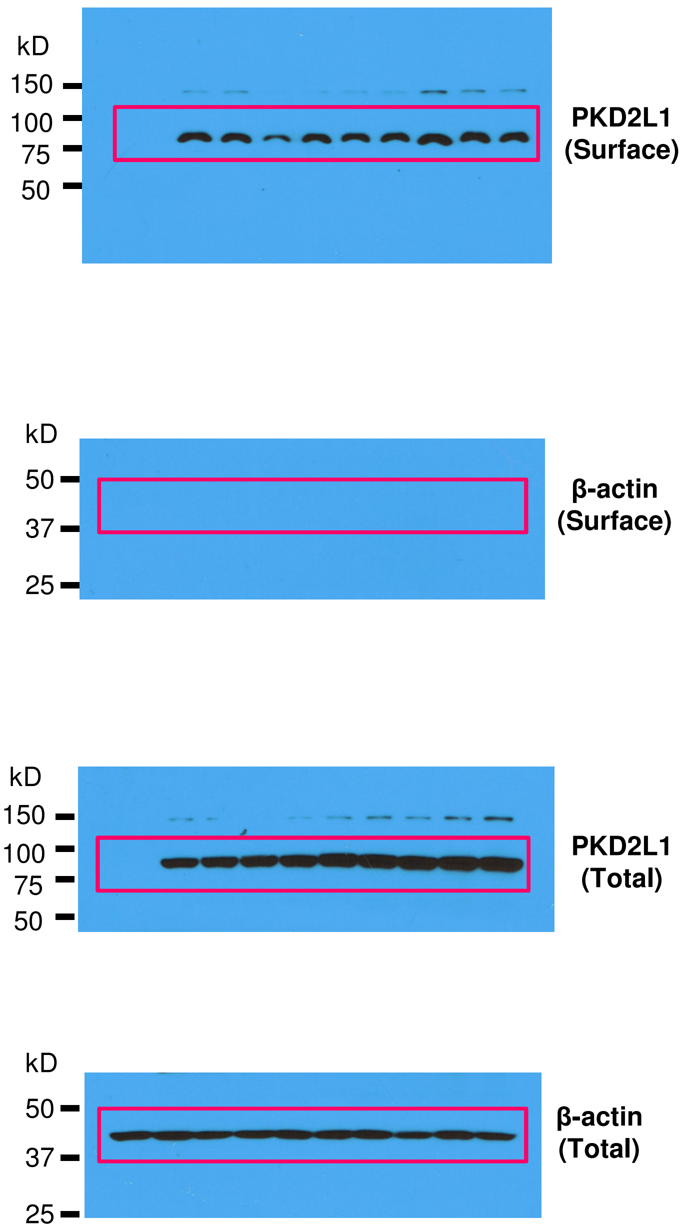

Figure 2e

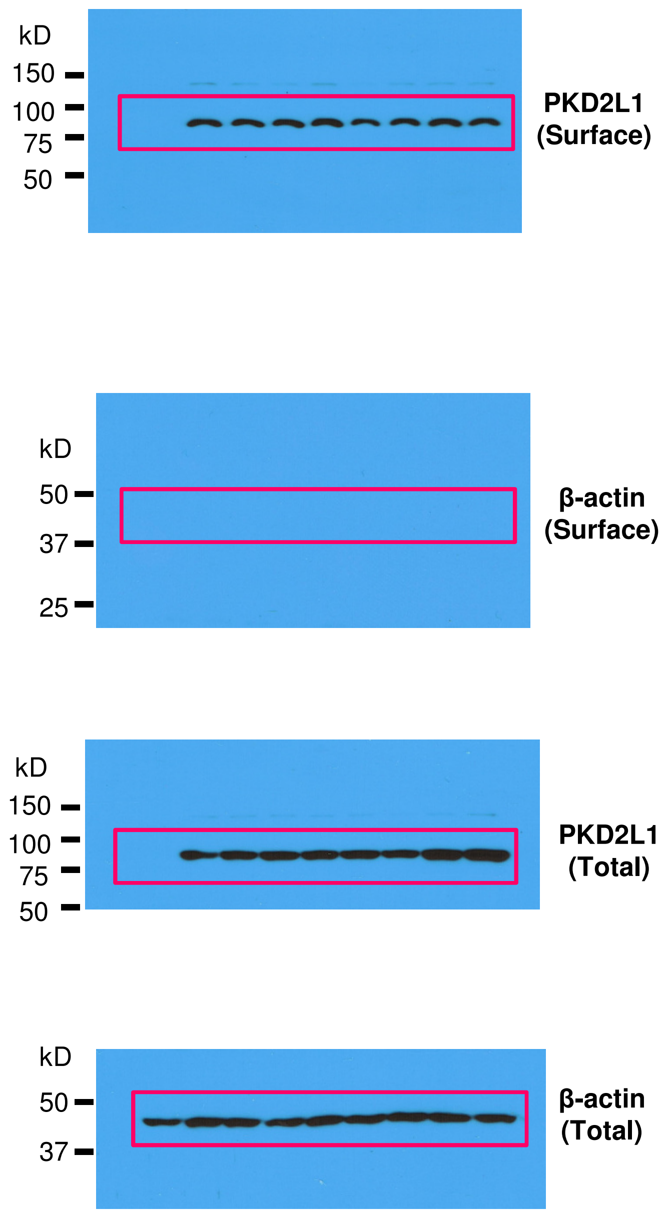

Supplementary Figure 12. Uncropped images of the original scans of immuoblots. Uncropped, full-size scans of immunoblots shown in Fig. 1e and Fig. 2e.

Figure 3c

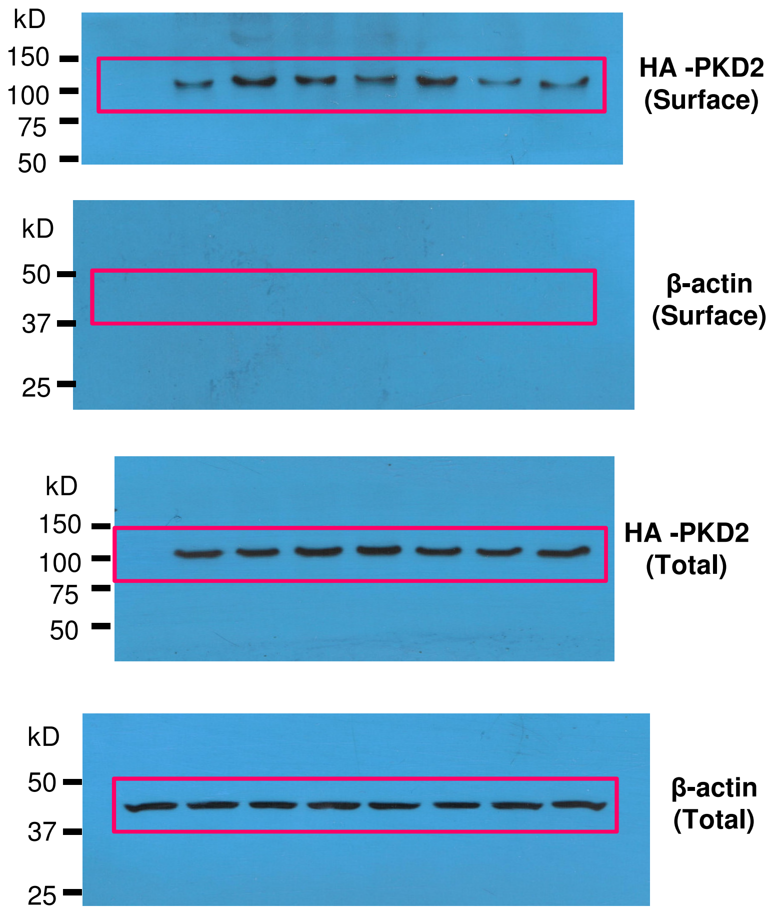

Figure 3d

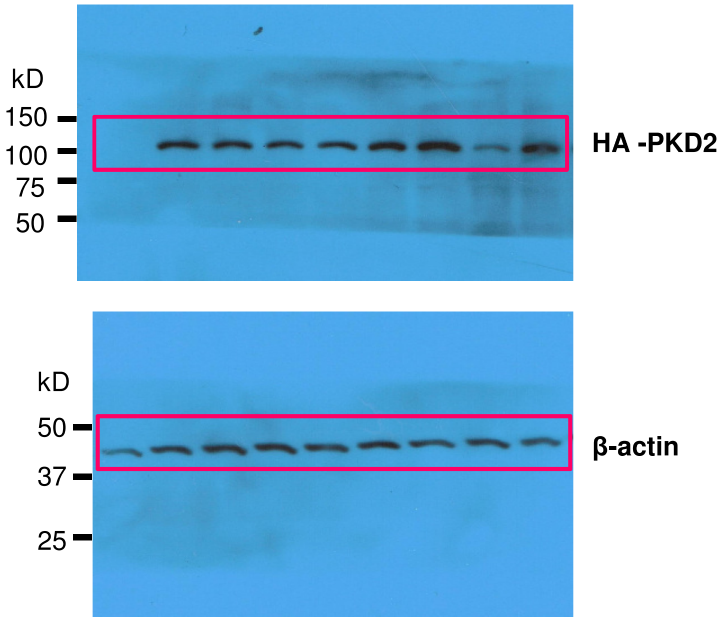

Supplementary Figure 13. Uncropped images of the original scans of immuoblots. Uncropped, full-size scans of immunoblots shown in Fig. 3c and d.

Supplementary Table 1. Cryo-EM data collection, refinement and validation statistics

	PKD2 F604P (EMDB-7786) (PDB-6D1W)
**Data collection/Processing**	
Voltage (kV)	300
Magnification	41,132
Defocus range (μm)	-0.6 – -2.4
Pixel size (Å)	1.2156
Total electron dose (e^-/Å^2)	80
Exposure time (s)	16
Number of images	1,992
Number of frames per image	80
Initial particle number	661,905
Final particle number	387,454
Resolution (unmasked, Å)	3.8
Resolution (masked, Å)	3.5
**Refinement and Validation**	
Number of atoms	14,452
R.M.S deviation	
Bond length (Å)	0.008
Bond angles (°)	0.794
Ramachandran	
Favored (%)	93.3%
Allowed (%)	6.5%
Outlier (%)	0.2%
Molprobity score	2.20

Supplementary Table 2. Primers used in this study

Mutation	Direction	Primer (5'-3')
**PKD2L1**		
L552N	Forward (F)	CGTCTTCTTCGTGAACCTGAACATGTTCC
	Reverse (R)	GGAACATGTTCAGGTTCACGAAGAAGACG
L553N	F	GTCTTCTTCGTGCTCAATAACATGTTCCTGGC
	R	GCCAGGAACATGTTATTGAGCACGAAGAAGAC
M555N	F	CGTGCTCCTGAACAATTTCCTGGCCATCATC
	R	GATGATGGCCAGGAAATTGTTCAGGAGCACG
F556N	F	GCTCCTGAACATGAACCTGGCCATCATCAA
	R	TTGATGATGGCCAGGTTCATGTTCAGGAGC
L557N	F	GCTCCTGAACATGTTCAATGCCATCATCAATGACAC
	R	GTGTCATTGATGATGGCATTGAACATGTTCAGGAGC
A558N	F	GAACATGTTCCTGAACATCATCAATGACAC
	R	GTGTCATTGATGATGTTCAGGAACATGTTC
I559N	F	GTTCCTGGCCAACATCAATGACACATATTC
	R	GAATATGTGTCATTGATGTTGGCCAGGAAC
I560N	F	CCTGGCCATCAACAATGACACATATTC
	R	GAATATGTGTCATTGTTGATGGCCAGG
L557E	F	GCTCCTGAACATGTTCGAGGCCATCATCAATGACAC
	R	GTGTCATTGATGATGGCCTCGAACATGTTCAGGAGC
L557D	F	GCTCCTGAACATGTTCGATGCCATCATCAATGACAC
	R	GTGTCATTGATGATGGCATCGAACATGTTCAGGAGC
L557Q	F	GCTCCTGAACATGTTCCAGGCCATCATCAATGACAC
	R	GTGTCATTGATGATGGCCTGGAACATGTTCAGGAGC
L557S	F	GCTCCTGAACATGTTCAGTGCCATCATCAATGACAC
	R	GTGTCATTGATGATGGCACTGAACATGTTCAGGAGC
L557C	F	GCTCCTGAACATGTTCTGTGCCATCATCAATGACAC
	R	GTGTCATTGATGATGGCACAGAACATGTTCAGGAGC
L557T	F	GCTCCTGAACATGTTCACGGCCATCATCAATGACAC
	R	GTGTCATTGATGATGGCCGTGAACATGTTCAGGAGC
L557G	F	GCTCCTGAACATGTTCGGGGCCATCATCAATGACAC
	R	GTGTCATTGATGATGGCCCCGAACATGTTCAGGAGC
L557A	F	GCTCCTGAACATGTTCGCGGCCATCATCAATGACAC
	R	GTGTCATTGATGATGGCCGCGAACATGTTCAGGAGC
L557V	F	GCTCCTGAACATGTTCGTGGCCATCATCAATGACAC
	R	GTGTCATTGATGATGGCCACGAACATGTTCAGGAGC
L557I	F	GCTCCTGAACATGTTCATTGCCATCATCAATGACAC
	R	GTGTCATTGATGATGGCAATGAACATGTTCAGGAGC
L557F	F	GCTCCTGAACATGTTCTTTGCCATCATCAATGACAC
	R	GTGTCATTGATGATGGCAAAGAACATGTTCAGGAGC
L557W	F	GCTCCTGAACATGTTCTGGGCCATCATCAATGACAC
	R	GTGTCATTGATGATGGCCCAGAACATGTTCAGGAGC
A558Q	F	GAACATGTTCCTGCAAATCATCAATGACAC
	R	GTGTCATTGATGATTTGCAGGAACATGTTC
A558S	F	GAACATGTTCCTGTCCATCATCAATGACAC
	R	GTGTCATTGATGATGGACAGGAACATGTTC

A558C	F	GAACATGTTCCTGTGCATCATCAATGACAC
	R	GTGTCATTGATGATGCACAGGAACATGTTC
A558T	F	GAACATGTTCCTGACCATCATCAATGACAC
	R	GTGTCATTGATGATGGTCAGGAACATGTTC
A558G	F	GAACATGTTCCTGGGCATCATCAATGACAC
	R	GTGTCATTGATGATGCCCAGGAACATGTTC
A558V	F	GAACATGTTCCTGGTCATCATCAATGACAC
	R	GTGTCATTGATGATGACCAGGAACATGTTC
A558I	F	GAACATGTTCCTGATCATCATCAATGACAC
	R	GTGTCATTGATGATGATCAGGAACATGTTC
A558L	F	GAACATGTTCCTGCTCATCATCAATGACAC
	R	GTGTCATTGATGATGAGCAGGAACATGTTC
A558F	F	GAACATGTTCCTGTTCATCATCAATGACAC
	R	GTGTCATTGATGATGAACAGGAACATGTTC
A558W	F	GAACATGTTCCTGTGGATCATCAATGACAC
	R	GTGTCATTGATGATCCACAGGAACATGTTC
L557W/A558N	F	CCTGAACATGTTCTGGAACATCATCAATGACAC
	R	GTGTCATTGATGATGTTCCAGAACATGTTCAGG
A558W/L557N	F	CCTGAACATGTTCAACTGGATCATCAATGACAC
	R	GTGTCATTGATGATCCAGTTGAACATGTTCAGG
K461A	F	CATCAGCTTCAACGCAACCATGACCCAG
	R	CTGGGTCATGGTTGCGTTGAAGCTGATG
**PKD2**		
F676N	F	CTTCATTCTTTTGAATATGAATTTGGCTATCATCAATG
	R	CATTGATGATAGCCAAATTCATATTCAAAAGAATGAAG
L677N	F	CATTCTTTTGAATATGTTTAATGCTATCATCAATGATAC
	R	GTATCATTGATGATAGCATTAAACATATTCAAAAGAATG
A678N	F	CTTTTGAATATGTTTTTGAATATCATCAATGATACTTAC
	R	GTAAGTATCATTGATGATATTCAAAAACATATTCAAAAG
I679N	F	GAATATGTTTTTGGCTAACATCAATGATACTTAC
	R	GTAAGTATCATTGATGTTAGCCAAAAACATATTC
A678Q	F	CTTTTGAATATGTTTTTGCAAATCATCAATGATACTTAC
	R	GTAAGTATCATTGATGATTTGCAAAAACATATTCAAAAG
A678S	F	CTTTTGAATATGTTTTTGTCTATCATCAATGATACTTAC
	R	GTAAGTATCATTGATGATAGACAAAAACATATTCAAAAG
A678T	F	CTTTTGAATATGTTTTTGACTATCATCAATGATACTTAC
	R	GTAAGTATCATTGATGATAGTCAAAAACATATTCAAAAG
L677G	F	CATTCTTTTGAATATGTTTGGGGCTATCATCAATGATAC
	R	GTATCATTGATGATAGCCCCAAACATATTCAAAAGAATG
L677A	F	CATTCTTTTGAATATGTTTGCGGCTATCATCAATGATAC
	R	GTATCATTGATGATAGCCGCAAACATATTCAAAAGAATG
L677F	F	CATTCTTTTGAATATGTTTTTTGCTATCATCAATGATAC
	R	GTATCATTGATGATAGCAAAAAACATATTCAAAAGAATG
L677Y	F	CATTCTTTTGAATATGTTTTACGCTATCATCAATGATAC
	R	GTATCATTGATGATAGCGTAAAACATATTCAAAAGAATG
L677W	F	CATTCTTTTGAATATGTTTTGGGCTATCATCAATGATAC
	R	GTATCATTGATGATAGCCCAAAACATATTCAAAAGAATG
W201A	F	GGCTGCGAGGTCTCGCGGGAACAAGACTCATG
	R	CATGAGTCTTGTTCCCGCGAGACCTCGCAGCC

K688A	F	CTTACTCTGAAGTGGCATCTGACTTGGC
	R	GCCAAGTCAGATGCCACTTCAGAGTAAG
D511V	F	GTTTCTGGAATTGTCTGGTTGTTGTGATCGTTGTGC
	R	GCACAACGATCACAACAACCAGACAATTCCAGAAAC
ΔY684	F	CTATCATCAATGATACTTCTGAAGTGAAATCTGACT
	R	AGTCAGATTTCACTTCAGAAGTATCATTGATGATAG
Y684A	F	CTATCATCAATGATACTGCCTCTGAAGTGAAATCTGACT
	R	AGTCAGATTTCACTTCAGAGGCAGTATCATTGATGATAG
L677N/N681L	F	GTTTATTGCTATCATCCTTGATACTTACTCTG
	R	CAGAGTAAGTATCAAGGATGATAGCAATAAAC
R581A	F	CAAATTCATCAATTTTAACGCGACCATGAGCCAG
	R	CTGGCTCATGGTCGCGTTAAAATTGATGAATTTG
F676G	F	CTTCATTCTTTTGAATATGGGTTTGGCTATCATCAATG
	R	CATTGATGATAGCCAAACCCATATTCAAAAGAATGAAG
F676A	F	CTTCATTCTTTTGAATATGGCTTTGGCTATCATCAATG
	R	CATTGATGATAGCCAAAGCCATATTCAAAAGAATGAAG
F676Y	F	CTTCATTCTTTTGAATATGTATTTGGCTATCATCAATG
	R	CATTGATGATAGCCAAATACATATTCAAAAGAATGAAG
F676W	F	CTTCATTCTTTTGAATATGTGGTTGGCTATCATCAATGATAC
	R	GTATCATTGATGATAGCCAACCACATATTCAAAAGAATGAAG
L677N/F676G	F	CTTCATTCTTTTGAATATGGGTAACGCTATCATCAATG
	R	CATTGATGATAGCGTTACCCATATTCAAAAGAATGAAG
L677N/F676A	F	CTTCATTCTTTTGAATATGGCTAACGCTATCATCAATG
	R	CATTGATGATAGCGTTAGCCATATTCAAAAGAATGAAG
L677N/F676Y	F	CTTCATTCTTTTGAATATGTATAACGCTATCATCAATG
	R	CATTGATGATAGCGTTATACATATTCAAAAGAATGAAG
L677N/F676W	F	CTTCATTCTTTTGAATATGTGGAACGCTATCATCAATG
	R	CATTGATGATAGCGTTCCACATATTCAAAAGAATGAAG
